# Supplementary material for: Allopregnanolone Promotes Migration and Invasion of Human Glioblastoma Cells through the Protein Tyrosine Kinase c-Src Activation
Source: Int J Mol Sci. 2022 Apr 30;23(9):4996. doi: 10.3390/ijms23094996 (PMC9105169; doi:10.3390/ijms23094996)
Supplement: Supplementary file 1 [file ijms-23-04996-s001.zip › ijms-1701149-supplementary.pdf]

Allopregnanolone promotes migration and invasion of human glioblastoma cells through c-Src activation

Carmen J Zamora-Sánchez[1], Claudia Bello-Alvarez[1], Mauricio Rodríguez-Dorantes[2], and Ignacio Camacho-Arroyo[1],*

SUPPLEMENTARY FIGURES AND FIGURE LEGENDS

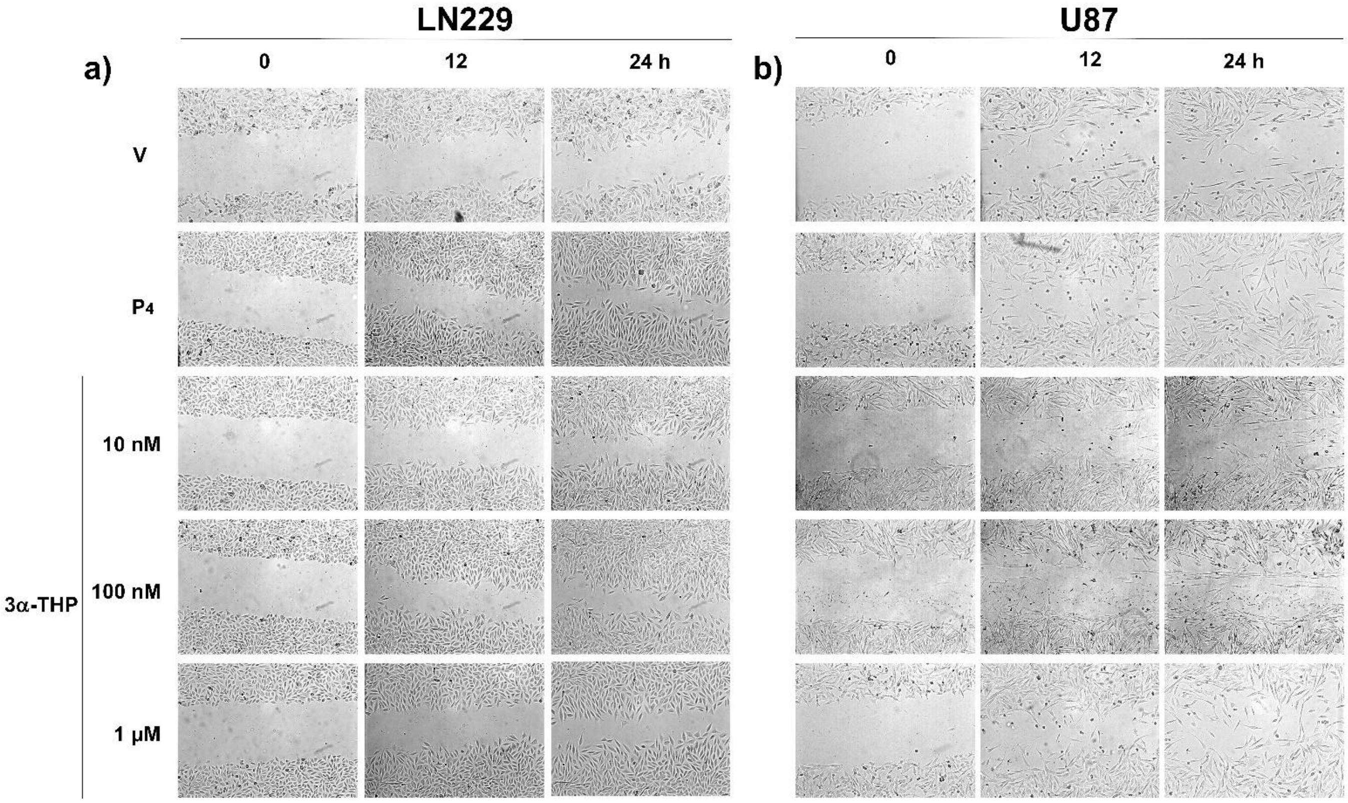

Supplementary Figure S1. Representative experiments of migration assays in **a)** LN229 and **b)** U87 cell lines. Cells were treated with progesterone (P4 10 nM) as a positive control and different concentrations of 3α-THP.

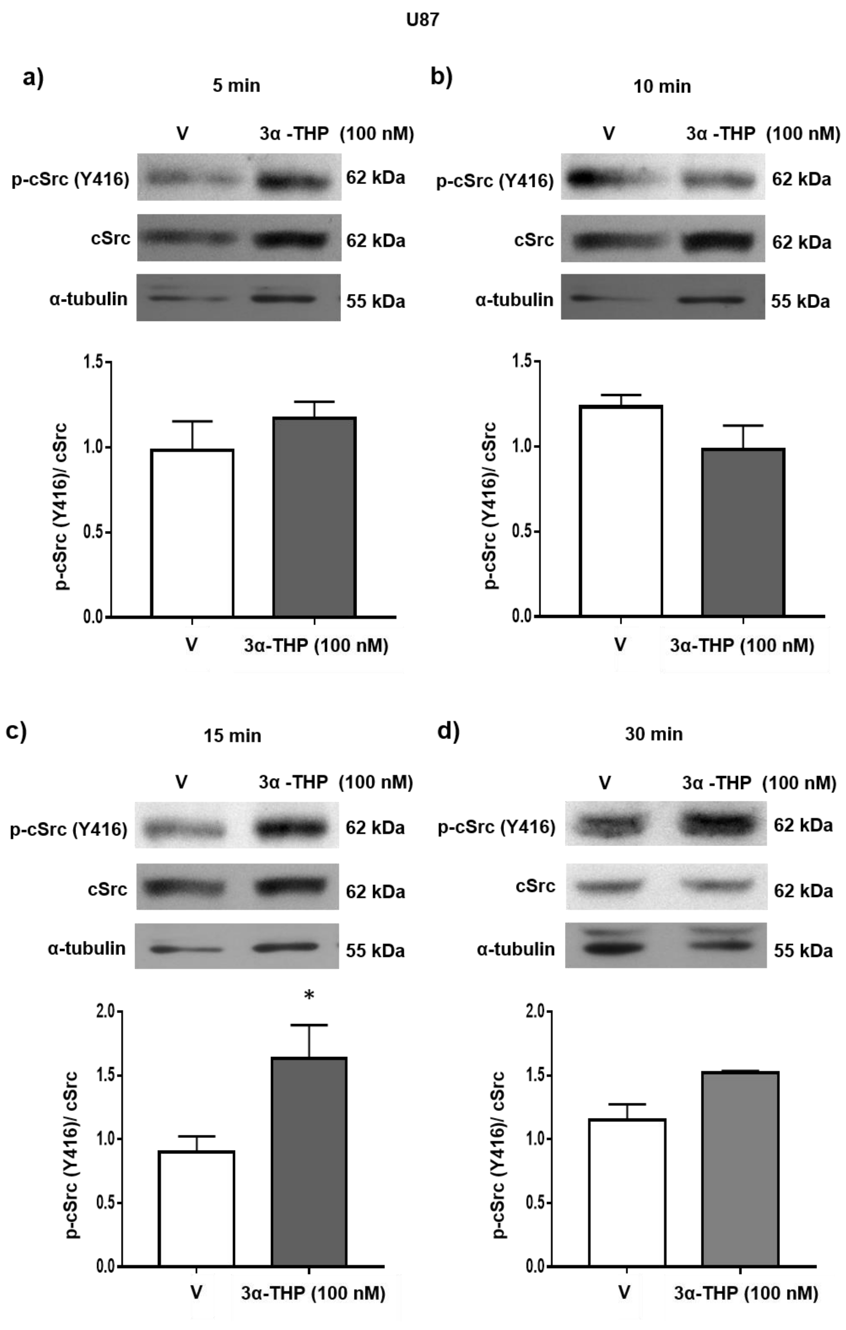

Supplementary Figure S2. Effect of 3α-THP on c-Src activation in U87 cells in a time-curse experiment. The phosphorylation of c-Src (Y416) was determined by Western blot under the treatment of 3α-THP 100 nM or Vehicle (V, EtOH 0.01 %) for **a)** 5 min; **b)** 10 min; **c)** 15 min; **d)** 30 min. The upper panel of each section shows a representative Western blot experiment of p-c-Src, total c-Src and α-tubulin as loading control. Each lower panel presents the densitometric analysis of p-cSrc/c-Src. Each column represents the mean ± SEM., n = 3; *$p < 0.05$ 3α-THP vs V.

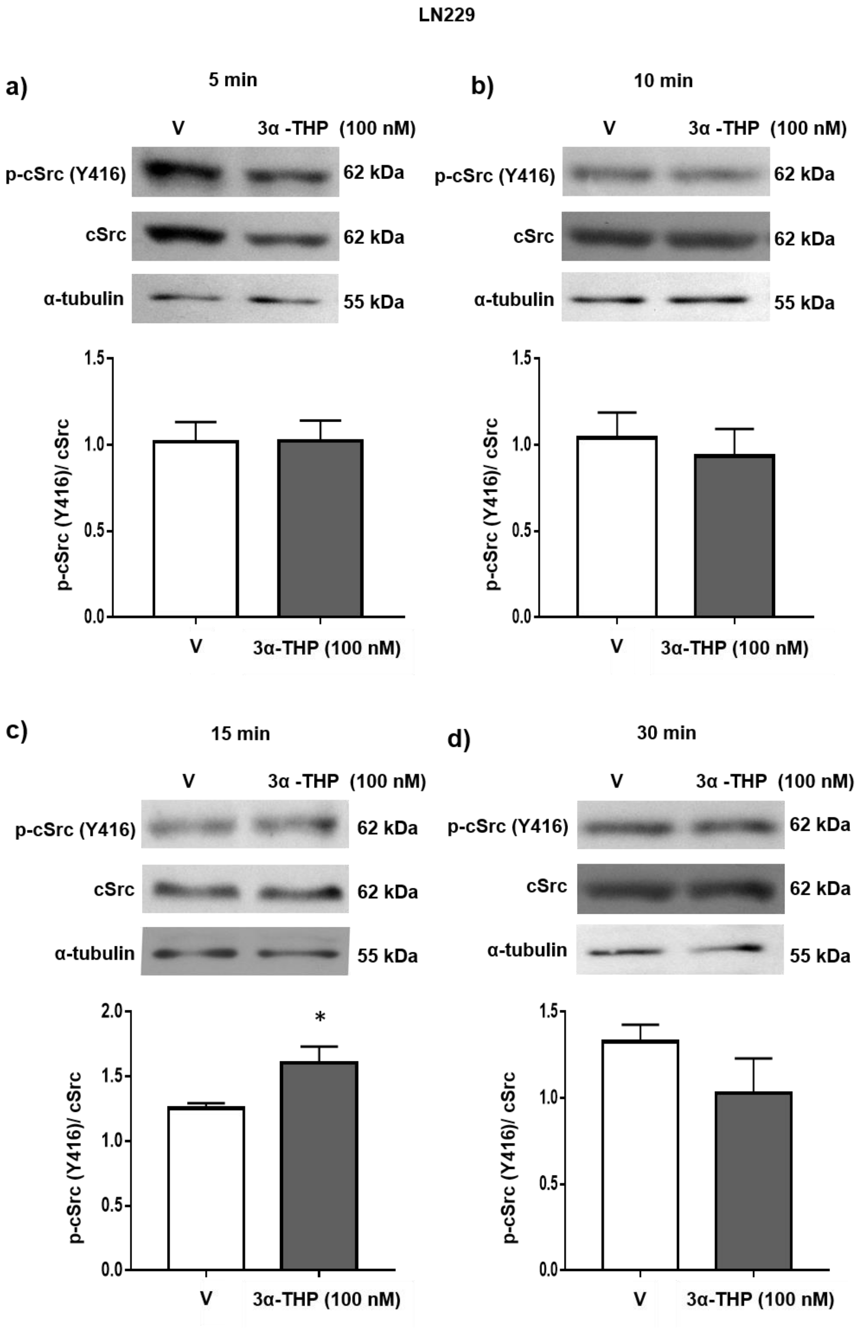

Supplementary Figure S3. Effect of 3α-THP on c-Src activation in LN229 cell line in a time-curse experiment. The phosphorylation of c-Src (Y416) was determined by Western blot under the treatment of 3α-THP 100 nM or Vehicle (V, EtOH 0.01 %) for **a)** 5 min; **b)** 10 min; **c)** 15 min; and **d)** 30 min. The upper panel of each section shows a representative Western blot experiment of p-cSrc, total c-Src and α-tubulin as loading control. Each lower panel presents the densitometric analysis of p-c-Src/c-Src. Each column represents the mean ± SEM., n = 3; *p <0.05 3α-THP vs V.

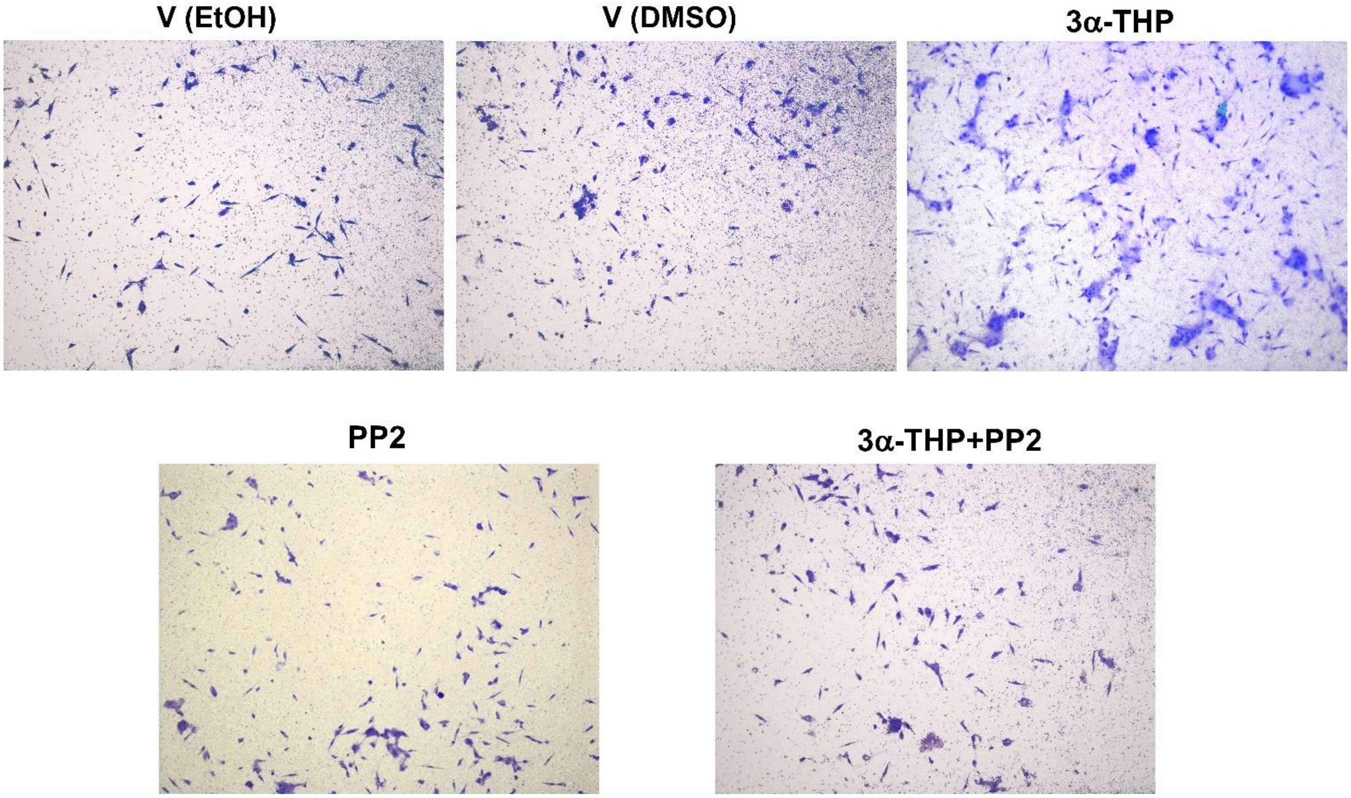

Supplementary Figure S4. Effect of 3α-THP and the pharmacological inhibitor of c-SRC, PP2 on U87 cell invasion. Representative images of invasion experiments in the U87 cell line. All photographs were taken at 10X augment.
